# ForCenS, a curated database of planktonic foraminifera census counts in marine surface sediment samples

**DOI:** 10.1038/sdata.2017.109

**Published:** 2017-08-22

**Authors:** Michael Siccha, Michal Kucera

**Affiliations:** 1 MARUM Center for Marine Environmental Sciences, University of Bremen, Leobener Straße 8, Bremen 28359, Germany

**Keywords:** Evolution, Palaeoceanography, Biooceanography

## Abstract

Census counts of marine microfossils in surface sediments represent an invaluable resource for paleoceanography and for the investigation of macroecological processes. A prerequisite for such applications is the provision of data syntheses for individual microfossil groups. Specific to such syntheses is the necessity of taxonomical harmonisation across the constituent datasets, coupled with dereplication of previous compilations. Both of these aspects require expert knowledge, but with increasing number of records involved in such syntheses, the application of expert knowledge via manual curation is not feasible. Here we present a synthesis of planktonic foraminifera census counts in surface sediment samples, which is taxonomically harmonised, dereplicated and treated for numerical and other inconsistencies. The data treatment is implemented as an objective and largely automated pipeline, allowing us to reduce the initial 6,984 records to 4,205 counts from unique sites and informative technical or true replicates. We provide the final product and document the procedure, which can be easily adopted for other microfossil data syntheses.

## Background & Summary

The composition of marine plankton communities reflects the properties of their surface-layer habitat^1^. In groups of plankton that produce fossils, information on community composition is preserved in marine sediments. Their fossil record can thus be used to reconstruct past surface-layer conditions. This procedure is contingent on the availability of observations on present-day communities, generated under the same conditions of spatiotemporal averaging and preservational bias as is the case for the fossil samples. This prerequisite is best met by census counts from surface sediment samples. Due to extensive efforts in exploration of the ocean floor, a large amount of observations on surface sediment properties, including composition of their constituent microfossil assemblages, have been generated. Such data have been used extensively in paleoceanography to calibrate assemblage compositions to surface ocean properties, in form of so-called transfer functions^2^, facilitating quantitative reconstructions of past ocean states.

The microfossil group with a particularly long history of usage in this regard are the planktonic foraminifera. Their shells can be easily identified to morphospecies level, are preserved in marine sediments across large parts of the world ocean and their species distribution shows a strong relationship to surface water hydrography^3^. Because of a distinct morphology that can be observed under standard stereomicroscopes combined with a limited diversity of the group, census counts of planktonic foraminifera can be generated with relatively little effort. As a result, planktonic foraminifera species distribution in surface sediments has been characterised in thousands of samples, collected by different methods. A standardisation of taxonomy of the group carried out within the CLIMAP project allowed the assembly of the first global calibration dataset^4^ (Data Citation 1), representing a substantial advance over pre-existing datasets generated by individual researchers often in a limited regional context^5^.

Since the pioneering effort of CLIMAP, data on planktonic foraminifera species abundance in surface sediments rapidly grew in number^6,7^ (Data Citation 2), facilitating the development of increasingly sophisticated transfer function approaches^8^. The data have been used to calibrate numerical models of foraminifera production^9^, explore their biodiversity patterns^10,11^ and develop methods to quantify carbonate dissolution on the sea floor^12^. With the accumulation of new data, the assembly and harmonisation of global datasets became increasingly difficult. Due to divergent taxonomic practice and human error during assembly of digital products, the latest global compilation^7^ suffers from internal inconsistency, uncontrolled duplication, and unsatisfactory documentation of taxonomic resolution. Since the release of the MARGO compilation, many new datasets have been generated and the taxonomy of the group has been better understood using molecular methods^13^, but no further systematic effort of data integration has been made.

Here we present the results of a long-term effort to provide a harmonised and consistent dataset of planktonic foraminifera census counts in surface sediment samples with curated taxonomy, accounting for different levels of taxonomic resolution. We explain and document the approach, which combines sequential dereplication of four previous syntheses, assembly of new data, and numerical and taxonomical treatment to achieve internal consistency. The resulting dataset is comprehensively commented for all modifications and includes the original data. The data-assembly procedure is objective and allows easy incorporation of new data. The final product provides an optimal starting point for the development of transfer functions, testing of ecological models and analyses of macroecological patterns. The approach itself can potentially guide similar efforts for other types of paleontological data syntheses.

## Methods

### Design of the analysis

Every effort in producing a globally harmonised synthesis of micropaleontological data will be confronted with three essential challenges. First, census counts generated by different authors across a considerable amount of time are not taxonomically harmonised. This problem arises because of inconsistent naming of the same taxonomic units (synonymy), inconsistent level of taxonomic resolution (splitting or lumping) and inconsistent documentation of the list of considered species (completeness). Second, in the presence of earlier compilation efforts, every subsequent data synthesis requires dereplication against earlier products. This is substantially complicated by the third challenge, which is the inconsistency in data and metadata recording. This leads to artificial inflation of the synthesis by ‘synonymous’ data entries that differ in aspects ranging from the syntax of the site identifiers, over composition differences resulting from rounding, to seemingly inexplicable differences in data associated with identical site identifiers. In our approach, we attempted to address all of these issues objectively.

To assemble the dataset, we decided to restrict the synthesis to data generated by the CLIMAP methodology, involving counting of about 300 specimens of planktonic foraminifera in the size class larger 150 μm. We considered four existing key compilations, the dataset of CLIMAP^4^ (Data Citation 1), the Brown University Foraminiferal Database (BUFD) (Data Citation 2), the ATL947 database^6^ (Data Citation 3) and MARGO^7^ (Data Citations 4–7). In addition, we searched the PANGAEA and NOAA paleoclimatology data repositories for datasets not incorporated in these and added those to the synthesis. In a first step, the taxonomy was manually standardised to a list of categories, following Hemleben *et al.*^14^ with modifications by Morard *et al.*^13^. For all included samples, metadata catalogues have been standardised and missing data were complemented from original publications as far as possible. New metadata categories were added to flag the discovered inconsistencies, facilitate reconstitution of the four main constituent datasets and allow geographical subdivision.

The following steps involved standardisation and correction of the counts. Deviations from the expected sum of constituent categories within a sample may occur because of rounding errors for relative abundances but also due to human error during digital data input or during cloning from earlier databases. Consequently, samples of insufficient quality were flagged and excluded from further processing. This concerned samples with severely inconsistent sum of categories, small sample size, samples taken using inappropriate sampling device, samples where too many specimens were left unidentified and samples with assemblage composition that is at odds with the known endemism pattern in modern planktonic foraminifera. The census counts in those samples are deemed likely to deviate from what would be expected for the sampling location for reasons other than the already considerable variability imposed by the spatial inhomogeneity of marine plankton flux. For example, census counts from plankton tows or sediment traps have been excluded because they do not account for the integration of seasonal and interannual variability in sedimentary assemblages.

The remaining samples were subjected to a dereplication procedure, the identification and treatment of duplicates (multiples) in the dataset, a key motivation for the analysis. It was carried out individually for each dataset and then sequentially to account for the known order of cloning among the four key datasets. Because the counts and metadata suffer from rounding errors and human error and the names (labels) for identical samples vary between compilations, a detection of duplicates is non-trivial. We used a series of conservative criteria avoiding the loss of potentially informative samples, such as technical replicates (the same assemblage of foraminifera counted twice by different taxonomists) and true replicates (multiple samples taken from the same location, even with the same device—such as different multicorer tubes, and counted independently). Counts and metadata in samples identified as duplicates were merged such that the retained sample contained the maximum amount of information. All steps and decisions were recorded and the data can be recovered at any stage of processing.

Outliers, samples that significantly differ in their assemblage composition from samples in their immediate surrounding, are not considered by our procedures. Outliers can result from plain errors (typographic errors, swapped latitude and longitude, etc.) taxonomic inconsistency among researchers or post-depositional processes such as dissolution of fragile tests at depth below the lysocline. Whilst the latter process can be objectively quantified and used to exclude samples, the other remain largely subjective. As a result, an outlier treatment cannot be implemented entirely objectively without a knowledge of the purpose of the intended analysis and we therefore leave this aspect of data processing to future users.

### Data sources

The compilation is based on all planktonic foraminifera assemblage count data from surface sediment samples in the size class larger 150 μm that we could identify in the PANGAEA and NOAA Paleoclimatology data repositories. The search was carried out on October 1st 2016, using search strings combining <planktic, planktonic, foraminifer*, census, assemblage, faunal distribution, counts>. The outcome was filtered to include only datasets of census counts in recent surface sediments. The data comprise the compilations of CLIMAP (Data Citation 1), the Brown University Foraminiferal Database (BUFD) (Data Citation 2), the ATL947 database (Data Citation 3) and MARGO (Data Citation 4–7) as well as the individual datasets of Huels *et al.* (Data Citation 8), Mohtadi *et al.* (Data Citation 9 and Data Citation 10), Salgueiro *et al.* (Data Citation 11), Siccha *et al.* (Data Citation 12) and Munz *et al.* (Data Citation 13). Also found but not included in the new compilation were the datasets of Cortese *et al.* (Data Citation 14) and Haddam *et al.* (Data Citation 15), the reasons are summarized in the section *Technical Validation*. All individual datasets found were of a later publication date than the MARGO database, except the dataset by Huels *et al.* (Data Citation 8). The data by Mohtadi *et al.* (Data Citation 9) were complemented to include counts of all species, following personal communication with the author. In the case of Munz *et al.* (Data Citation 13) the complete count data was obtained by personal communication and used instead of the published version in PANGAEA. The new datasets were merged and labelled as ‘Additions’ in tables and figures of this study.

### Taxonomic standardization

Taxonomic standardization was performed individually on all datasets. Data in their original taxonomic form (i.e., with uncorrected taxonomy) are only available through access to their original repository (see data citations below). The harmonised taxonomy as applied in this analysis follows Hemleben *et al.*^14^ as implemented in Morard *et al.*^13^ and expanded by Weiner *et al.*^15^ and Spezzaferri *et al.*^16^. The taxonomic list we use comprises 47 species categories, three multi-species categories and six sub-species (morphospecies) categories (Table 1 (available online only)). Six of the 47 species categories have no entries as the abundances of the respective species have not been recorded so far. Of these, four categories refer to species that are too small to be recorded in the analysed size fraction and two categories (*G. elongatus*, *G. radians*) have only recently been established during taxonomic revisions^15,17^ and have thus not been counted before. Synonymy has been resolved manually as documented in Table 2. All synonyms could be unambiguously assigned to categories in Table 2 except for the cases described below.

In the CLIMAP database, following the BUFD (Data Citation 2) and ATL947 (Data Citation 3) compilations the category ‘G*. pachyderma*’ was interpreted as ‘P/D intergrades’. This category was then merged with the ‘*N. pachyderma d*’ category into the category ‘*N. incompta*’. The category *G. flexuosa* was removed and the abundances of this category merged with the category *G. menardii*. This is because there is no evidence for the morphotype represented by *G. flexuosa* being a separate species^13^. In the ATL947 database the category ‘P/D intergrades’ was removed and the abundances of this category merged with the category *N. incompta*. The justification for this decision is documented in the MARGO synthesis^7^. In the Brown University Foraminiferal Database, the category *G. flexuosa* was removed and the abundances of this category were merged with the category *G. menardii.* The category *G. crassula* was removed and the abundances of this category merged with the category ‘unidentified’. The species *G. crassula* appears to be extinct^18^ but its morphology cannot be unambiguously linked to an extant species, making it impossible to decide to which of the known species the counts for this category should be assigned. The category ‘other identified’ was merged with the category ‘unidentified’. In the MARGO database the category ‘P/D intergrades’ was removed and the abundances of this category was merged with the category *N. incompta*. The category *G. crassula* was removed and the abundances of this category merged with the category ‘unidentified’. The category ‘other identified’ was merged with the category ‘unidentified’. For the dataset of Munz *et al.* (Data Citation 13) we could obtain the original raw count data, which includes more categories than the version with relative abundances published via PANGAEA. In the raw data the category *G. puncticulata* was removed and the abundances of this category merged with the category *G. inflata. Globorotalia puncticulata* is an extinct species^19^, but its morphology is partly overlapping with that of its descendant *G. inflata*. The category ‘P/D intergrades’ was removed and the abundances of this category was merged with the category *N. incompta*.

The datasets of CLIMAP, BUFD and ATL947 were compiled in such a way that their constituent taxonomic categories are resolved for all records. The most comprehensive species list in the CLIMAP dataset contains 37 unique categories common with Table 3. The remaining four categories included in Table 1 (available online only) but not in CLIMAP refer mostly to small and rare species. Rather than setting their abundances to zero artificially, we have labelled these as ‘not available’, realising that it the vast majority of the cases the observed abundances would have been zero. In several studies among the Additions, the taxonomic resolution was not sufficiently clearly documented (Table 3). Notably, the species lists in Mohtadi *et al.* (Data Citations 9 and 10) and Salgueiro *et al.* (Data Citation 11) contain about 1/3 fewer categories than the average of other studies. These datasets are regionally constrained and it is likely that they only reported species that were abundant in the studied region. This is confirmed by an inspection of the methods description in Salgueiro *et al.*^20^, who mention the occurrence of rare species like *G. crassaformis* in the paper, but the category is not provided in the data file (Data Citation 11). Therefore, we assigned the value of ‘not available’ to all entries for categories not included in the data file for a given study.

In cases where the original taxonomy admitted lumping of species, we retained these categories as multi-species categories. This applies for example to *G. ruber* as a sum of *G. ruber* pink and white in the Mediterranean (Data Citation 7). In these cases, the constituent categories could be unambiguously identified as not available. In addition to formally described species, we retained in the counts the separation of distinct morphotypes. Even though these likely do not represent different species, their abundance has been frequently and consistently recorded. This applies specifically to the separation of *T. sacculifer* and *T. trilobus*, *G. truncatulinoides* sinistral and dextral and *T. quinqueloba* sinistral and dextral. Where separate counts are available, these are included under the label ‘morphotypes’ of the recognised species. This approach provides flexibility to accommodate future taxonomic revisions.

### Metadata standardization and addition of descriptors

Metadata standardization was also performed individually on the individual datasets (Table 4). The unit of the variable ‘Sample_depth’ was standardized to meters. Entries of zero in ‘Sample_depth_lower’ were corrected into entries of zero for ‘Sample_depth_upper’. The variable ‘Sample_depth_average’ was calculated where possible. Entries in the variable ‘Device’ were standardized into the categories ‘Piston’, ‘Gravity’, ‘Trigger’, ‘Grab’, ‘Giant Box’, ‘Box’, ‘Multi’ and ‘CTD’ where applicable. All entries in the variable ‘Sample_name’ were transformed to uppercase. We realise that in many cases the pattern of capitalisation of the names has a meaning, but we note that capitalisation has been used so inconsistently, that it is not possible to reconstitute it. Ignoring capitalisation makes automated processing of names much more tractable. Missing metadata in the input data was completed by searching the original publications where possible. The variable ‘Count_min’ denoting the minimum number of counted individuals was introduced and populated with information where available. In case where no information was available, the value was set to the common standard of 300 counted individuals. Detailed information on the original publication was added in form of the variables ‘Author’, ‘Journal’, ‘Year’ and ‘Publication_doi’. Three binary flag variables were added to the metadata, ‘Error’, ‘Ocean’ and ‘Database’, describing the treatment of the data, the source of the count data and the oceanographic region of the sampling site, respectively (Tables 5,6,7). The binary flags are constructed in a way that their value can express any combination of the possible states. For example, a ‘Database’ flag value of 7 for a count would indicate the inclusion of the count in the CLIMAP, the BUFD and the ATL947 compilations (1+2+4).

### Standardization and correction of count data

All relative count data were standardized to the range of 0 to 1. The ‘total count’ of samples with absolute counts was corrected if it did not correspond to the sum of the categories. Many of the analysed counts have an explicitly mentioned category ‘unidentified’. In theory, where this category is given, and its value is zero, all of the categories not explicitly considered by such study could be set to zero. We assumed the rounding error of individual categories to be 0.1% and the average total rounding error E_r_ of a sample expressed as a fraction to be *R*_*r*_=(n*0.001)/2 with n being the number of given categories. Therefore, in case of the sum of all categories being within rounding errors (sum of relative categories >1-E_r_) of the given sum and where zero is given for the ‘unidentified’ category, the sample was assumed to be complete and all non-present categories were filled with zeros. The relative abundances for all samples were recalculated to sum up to 1, except for samples where the sum of relative categories deviated by more than 5% of the expected sum of 1, which were flagged (Table 5, ‘Error’ flag bit 7) and excluded from further analyses. All samples that had a total count below 150 individuals were also flagged (Table 5, ‘Error’ flag bit 8) and excluded from further analyses.

### Removal or correction of counts in records of insufficient taxonomical quality

All counts with entries in the ‘unidentified’ category larger than 5% were flagged (Table 5, ‘Error’ flag bit 6) and excluded from further analyses. Counts from the Pacific, Indian Ocean or Red Sea with relative abundances >1% in the category *G. ruber* pink were also flagged (Table 5, ‘Error’ flag bit 5) and excluded from further analyses. In samples from the Pacific, Indian Ocean or Red Sea with relative abundances <1% in the category *G. ruber* pink, the abundances of this category were merged with the category ‘unidentified’. All values in the merged category *G. ruber* pink and white from the Pacific, Indian Ocean or Red Sea were resolved into the category *G. ruber* white. The reason for this revision is the observation that *G. ruber* pink has been extinct in the Indopacific since the last Interglacial^21^ and recent genetic studies confirmed the endemicity of *G. ruber* pink in the Atlantic^13^. In addition, counts from the Atlantic and the Mediterranean Sea with relative abundances >1% in the categories *G. conglomerata* or *G. hexagonus* or *G. adamsi* were flagged (Table 5, ‘Error’ flag bit 5) and excluded from further analyses. In counts from the Atlantic or Mediterranean with relative abundances <1% in the categories *G. conglomerata* or *G. hexagonus* or *G. adamsi*, the abundances of these categories were merged with the category ‘unidentified’. This treatment reflects the known endemicity of these three species in the Indopacific region^14^.

### Removal of counts for other reasons

All records without geographical coordinates were flagged (Table 5, ‘Error’ flag bit 10) and excluded from further analyses. All counts obtained from samples that have been taken with a non-standard sampling device, being neither ‘Box’ or ‘Giant Box’ or ‘Piston’ or ‘Gravity’ or ‘Grab’ or ‘Trigger’ or ‘Multi’ or ‘Mini’ or ‘CTD’ were flagged (Table 5, ‘Error’ flag bit 9) and excluded from further analyses.

### Control for duplication and removal of duplicates

The identification of duplicates (multiples) in the dataset was one of the main motivations for the generation of the new database. The simple detection of identical samples with exactly the same name at exactly the same location with identical count data is not sufficient as the position and assemblage data suffer from rounding errors and human error and the sample names for identical samples vary between compilations. Initial tests revealed the presence of three different types of duplicates in the data: ‘plain duplicates’, samples with identical names located a short geographic distance apart, containing a highly similar species assemblage, ‘incorrect position duplicates’, samples with identical name containing a highly similar species assemblage, but potentially located far apart and ‘different name duplicates’, samples with different names but located close to each other and containing a highly similar species assemblage.

The automatic detection of duplicates was carried out using conservative criteria. Basic criteria for all types of duplication were a maximum deviation in counts of individual categories <1% ignoring categories with no information and a maximum deviation in total counted individuals of 3% (of the average of the total count value for the sample pair). Sample pairs (and multiples) satisfying these criteria were sequentially subjected to a test for one of the following additional criteria. For the case of a ‘plain duplicate’ the additional criteria were maximum geographical distance between the pair of samples shorter than 2.621 km (the distance between 0.5′N 0.5′W and 0.5′S 0.5′E across the equator) and an identical name (Levenshtein distance between sample names of zero). For the case of an ‘incorrect position duplicate’ the additional criterion was an identical name (Levenshtein distance between sample names of zero). Lastly, for the case of a ‘different name duplicate’ the additional criterion was a maximal geographical distance between samples shorter than 0.5242 km (the distance between 0.1′N 0.1′W and 0.1′S 0.1′E across the equator). Counts that satisfied any one of these criteria were collected in lists of ‘duplicates’ and treated and removed sequentially, that is the test for ‘incorrect position’ duplicates was only conducted after all ‘plain’ duplicates had been treated.

The existence of combinations of the three duplication reasons makes such duplicates particularly resistant to detection. Indeed, we identified cases where both the name and the geographical position were different beyond threshold, but the samples still could be identified as duplicates. (‘ELT44.27-PC’ is identical to ‘E44-27B’/‘M8_12-1’ is identical to ‘M8/12-1’/‘A260210A’ is identical to ‘AII-15-602-10A’). Therefore, we implemented a final manual step in the duplication control, where, after all other cases have been treated automatically, a new list of possible duplicates was generated using only the basic criteria of faunal similarity, and inspected by the compiler.

The obtained lists of duplicate samples were subjected to a merging procedure designed to retain a maximum of the available information of all the involved samples. If a pair or a multiple of samples were merged, assemblage data with the highest number of counted taxa was carried over to the new merged sample. The geographic position with a highest precision was assigned to the merged sample. In case of a set of ‘incorrect position duplicates’, the ‘correct’ position was determined by first checking whether the discrepancy in location is a result of incorrect transformation of the coordinates (decimals/minutes). To this end, the coordinates of the counts in question were transformed assuming that the decimal places were not fractions of degrees but untransformed minutes. All geographical distances for the combinations of transformed and untransformed coordinates (excluding combinations with more than two incorrectly transformed coordinates) were calculated and checked whether the transformation translocated the sample more than 3.7 km from the original position (an ill-transformation of at least 5’). If this conditions was met and the samples in question would come to lie within a distance of less than 2.621 km, the transformation was accepted and the samples considered as duplicates. If the screening for incorrectly transformed coordinates was negative, the ‘correct’ position was determined by cross checking with ETOPO1 (Data Citation 16). The position data of the sample whose given water depth matched best with the water depth for the respective position in the ETOPO1 data was carried over to the merged sample. In terms of sample depth in the sediment the most complete set of information was given precedence (upper and lower boundary and average available), if only one number was available precedence was given to the available average depth. For all other sample metadata precedence was given to the existence of information in contrast to no available information (e.g., Sampling device). In case of conflicting metadata, the data of the older publication was used.

## Data Records

The ForCenS dataset is published as a single tab-delimited text file (Data Citation 17). Sample metadata are stored in columns 1 to 21 as described in Table 4. Variable names are given in the first row, variable units in the second row. Three blocks of species categories (Table 1 (available online only)) abundance data follow the metadata: first the original data as found in the data sources, reformatted only taxonomically. Next are the absolute count data, where available, with applied corrections and modifications, where applicable. The last block represents the data expressed as relative proportions, with applied corrections and modifications where applicable. This type of data is provided for all records in the dataset. This dataset comprises all records included in the analysis. The users are advised that at the end of each block, six columns contain data on morphotype abundances, the sums of which are already included in their parent taxonomic category.

Using the flags defined in Tables 5,6,7, users can reduce the list to reconstitute any of the original datasets, exclude replicates or produce a regionally constrained dataset. For convenience a second data file is published as tab-delimited text file with the same metadata structure as above, but only including records passing all selection criteria (Error flag <=1) and showing only the relative abundances of species. Both datasets are available via PANGAEA (Data Citation 17). The current implementation of the PANGAEA data portal facilitates versioning of datasets. This means that any expansion of the synthesis with new, overlooked or previously unavailable records can be carried out by the authors following the procedure described in this contribution and then uploaded as a new version.

## Technical Validation

**Figure 1:**
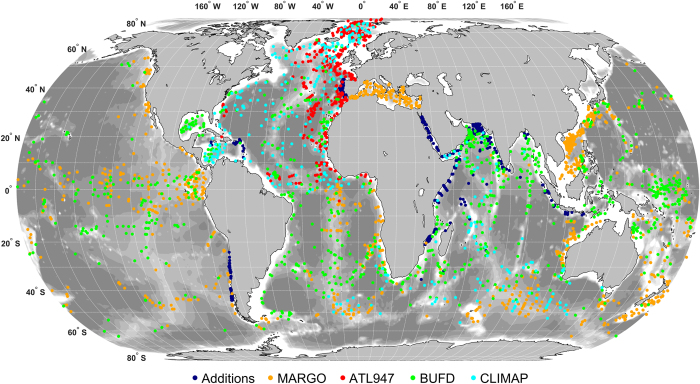
Location of all census counts retained in the ForCenS compilation. Colours denote the sample source, the first occurrence of a sample in a compilation taking precedence over reuse in later compilations.

**Figure 2:**
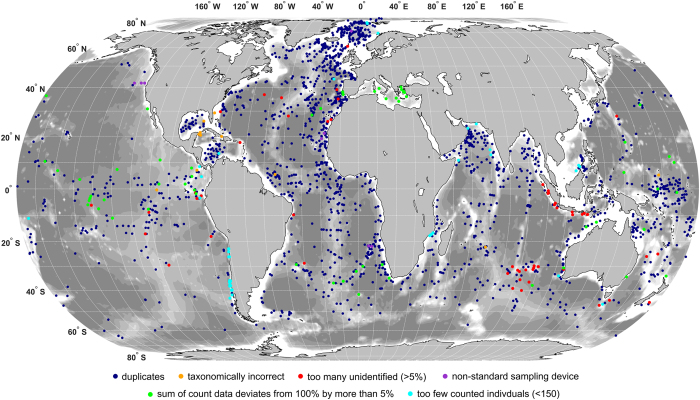
Location of all census counts excluded from the ForCenS compilation with colours denoting the reason of exclusion.

The initially assembled dataset including four previous compilations and six new datasets contained 6,984 census counts (Table 8). The initial processing (Table 9) excluded 229 counts, the majority for numerical reasons (53.7% of the excluded cases) or taxonomical issues (37.6%). The subsequent dereplication of the individual datasets showed that the MARGO database held 486 internal duplicates (12.9% of the samples). These duplicates were included intentionally in the MARGO datasets as outgroups for the regional calibration datasets, e.g., some samples from the Atlantic were included in the Indian Ocean and the Mediterranean datasets and there was an intentional overlap across the tropics between the South and North Atlantic datasets (Data Citation 4). The six new datasets contained 624 census counts, of which 56 were excluded during initial processing, no internal duplicates were found. The final processed and dereplicated ForCenS database (Data Citation 17) comprises 4,205 singular census counts (Table 8, Fig. 1). The distribution of all excluded samples, with identification of the reason for exclusion is shown in Fig. 2.

In its present form, ForCenS (Data Citation 17)contains not only counts from unique sites but also a small number of informative technical or true replicates. To illustrate the origin of these replicates, we provide an example. The original CLIMAP dataset contains 375 counts of which the initial processing retained 351 counts. After the sequential dereplication procedure up to and including the ATL947 dataset the database contained 492 counts with ‘CLIMAP Projects members’ in the ‘Author’ metadata category, an inflation by 141 counts. This inflation occurs already in the individual compilations and is not the result of our dereplication. For example, there are 212 counts from the Atlantic in the original CLIMAP database, but 266 counts attributed to CLIMAP were found in ATL947. Individual inspection of count pairs with identical name between the CLIMAP and ATL947 datasets reveals that they differed significantly in assemblage composition and were therefore not recognized as duplicates during our dereplication procedure. Although the reason is not mentioned in the original publications by Pflaumann *et al.*^6,22^, we conclude that the inflation is the result of recounting of the same samples (probably to check for taxonomic consistency) and the samples are correctly retained because they represent informative technical replicates.

Cortese *et al.*^23^ published a compilation of planktonic foraminifera census counts containing 1,223 samples, which was based on a previous compilation by Crundwell *et al.*^24^, the MARGO Indo-Pacific dataset (Data Citation 6), Mohtadi *et al.* (Data Citations 9 and 10) and additional unpublished data of Crundwell. The dataset of Crundwell *et al.*^24^ has not been published; but it was reported to consist of 891 samples of which all except for 24 samples were taken from datasets that were included in the MARGO compilation^7^. Therefore, we could establish that in theory, a dereplication of Cortese dataset (Data Citation 14) with the ForCenS dataset should have led to an addition of a maximum of 230 samples (assuming that only a single sample of the MARGO Indo-Pacific dataset (Data Citation 6) was added and all other were unpublished data). However, our dereplication procedure retained 427 samples of the Cortese dataset (Data Citation 14) (before manual dereplication), most of which had a partner with the same name in the MARGO dataset^7^ but had a significantly different assemblage composition from this partner sample. As no modifications to the data from the MARGO compilation^7^ were mentioned in the publication of Cortese *et al.*^23^, and we cannot reconstruct the reason for the differences in the data for apparently identical samples, we chose to exclude the full dataset even though several unique and valuable new census counts must have been included.

A similar situation occurred during the processing of the dataset published by Haddam *et al.*^25^ In contrast to the Cortese dataset (Data Citation 14) the dataset of Haddam (Data Citation 15) is annotated with the source of the individual census count. Amongst the 598 samples in the dataset, 125 are annotated with ‘French database, unpublished’, the remainder are labelled with either ‘MARGO database’ or ‘Cortese database’. In an attempt to avoid the problems that occurred with the addition of the Cortese dataset (Data Citation 14) to our compilation, we reduced the dataset of Haddam (Data Citation 15) to the 125 samples labelled as unpublished before processing. The sequential dereplication procedure retained only 45 out of these 125 samples as unique (before the manual dereplication step). Again, many of the retained samples have the same name as a sample from the MARGO dataset^7^ but a significantly different assemblage composition. Among the samples labelled ‘French database, unpublished’, many were identified to occur amongst the oldest census counts included in the initial CLIMAP (Data Citation 1) compilation. We can exclude the possibility of recounts (technical replicates) as the census counts are identical. Because we were unable to unambiguously identify which census counts in the dataset of Haddam (Data Citation 15) were unique, the dataset was excluded from the ForCenS compilation.

## Additional Information

**How to cite this article:** Siccha, M. & Kucera, M. ForCenS, a curated database of planktonic foraminifera census counts in marine surface sediment samples. *Sci. Data* 4:170109 doi: 10.1038/sdata.2017.109 (2017).

**Publisher’s note:** Springer Nature remains neutral with regard to jurisdictional claims in published maps and institutional affiliations.

## Supplementary Material



## Figures and Tables

**Table 1:** Taxonomic categories considered in the ForCenS database.

**No**	**Genus**	**Species**
	Multi-species categories	
1	*Dentigloborotalia*	*anfracta*
2	*Globorotalia*	*cavernula*
3	*Globorotalia*	*crassaformis*
4	*Globorotalia*	*hirsuta*
5	*Globorotalia*	*menardii*
6	*Globorotalia*	*scitula*
7	*Globorotalia*	*truncatulinoides*
8	*Globorotalia*	*tumida*
9	*Globorotalia*	*ungulata*
10	*Globorotalia*	*theyeri*
11	*Globoconella*	*inflata*
12	*Neogloboquadrina*	*dutertrei*
13	*Neogloboquadrina*	*incompta*
14	*Neogloboquadrina*	*pachyderma*
15	*Pulleniatina*	*obliquiloculata*
16	*Globoquadrina*	*conglomerata*
17	*Globorotaloides*	*hexagonus*
18	*Berggrenia*	*pumilio*
19	*Globigerina*	*bulloides*
20	*Globigerina*	*falconensis*
21	*Globigerinoides*	*conglobatus*
22	*Globigerinoides*	*ruber* (pink)
23	*Globigerinoides*	*ruber* (white)
24	*Trilobatus*	*sacculifer*
25	*Globigerinoides*	*tenellus*
26	*Orbulina*	*universa*
27	*Beella*	*digitata*
28	*Globigerinella*	*siphonifera*
29	*Globigerinella*	*calida*
30	*Globigerinella*	*adamsi*
31	*Turborotalita*	*quinqueloba*
32	*Turborotalita*	*humilis*
33	*Globoturborotalita*	*rubescens*
34	*Sphaeroidinella*	*dehiscens*
35	*Candeina*	*nitida*
36	*Globigerinita*	*glutinata*
37	*Globigerinita*	*uvula*
38	*Globigerinita*	*minuta*
39	*Tenuitella*	*iota*
40	*Hastigerina*	*pelagica*
41	*Hastigerinella*	*digitata*
	not present in ForCenS	
42	*Globorotaloides*	*elongatus*
43	*Globigerinella*	*radians*
44	*Orcadia*	*riedeli*
45	*Turborotalita*	*clarkei*
46	*Tenuitella*	*fleisheri*
47	*Tenuitella*	*parkerae*
	Multi-species categories	
	*G. menardii* (**5**) & *G. tumida* (**8**)	
	*G. ruber* (pink) **(22)** & *G. ruber (white)* **(23)**	
	*T. humilis* **(32)** & *B. pumilio **(18)***	
	Morphotype categories	
24 A	*T. sacculifer* with sac-like chamber	
24 B	*T. sacculifer* without sac-like chamber	
7 C	*G. truncatulinoides* dextral coiling	
7 D	*G. truncatulinoides* sinistral coiling	
31 C	*T. quinqueloba* dextral coiling	
31 D	*T. quinqueloba* sinistral coiling	

**Table 2:** Synonymisation used in preparing the ForCenS database.

**ForCenS species**	**synonyms**
*Beella digitata*	*Globigerina digitata*
	*Globigerinella digitata*
*Berggrenia pumilio*	*Globigerinita pumilio*
*Dentigloborotalia anfracta*	*Dentagloborotalia anfracta*
	*Globorotalia anfracta*
*Globigerinella calida*	*Globigerina calida*
*Globigerinella siphonifera*	*Globigerinella aequilateralis*
*Globigerinita uvula*	*Globigerinita bradyi*
*Globigerinoides tenellus*	*Globoturborotalita tenella*
*Globoconella inflata*	*Globorotalia inflata*
*Globorotalia menardii*	*Globorotalia menardii flexuosa*
*Globorotalia tumida*	*Globorotalia tumida flexuosa*
*Globorotaloides hexagonus*	*Globoquadrina hexagona*
*Globoturborotalita rubescens*	*Globigerina rubescens*
*Hastigerinella digitata*	*Hastigerina digitata*
*Neogloboquadrina pachyderma*	*Globigerina pachyderma*
	*Neogloboquadrina pachyderma* sinistral
*Neogloboquadrina dutertrei*	*Globoquadrina dutertrei*
*Neogloboquadrina incompta*	*Neogloboquadrina pachyderma* dextral
*Tenuitella iota*	*Globigerinita iota*
*Trilobatus sacculifer*	*Globigerinoides sacculifer*
	*Globigerinoides trilobus*
*Turborotalita humilis*	*Globigerina humilis*
	*Turborotalia cristata*
	*Turborotalia humilis*
*Turborotalita quinqueloba*	*Globigerina quinqueloba*
	*Turborotalia quinqueloba*

**Table 3:** Details of the constituent datasets of the ForCenS database.

**Dataset**	**Year**	**Entries**	**Species**	**Categories**	**Reference**
CLIMAP	1981	375	37	44	26
BUFD	1999	1,265	36	43	27
ATL947	2003	947	31	39	28
MARGO	2005	3,773	39	49	19–32
Huels	1999	21	30	34	33
Mohtadi	2005	91	20	22	34
Mohtadi	2007	34	18	20	35
Salgueiro	2008	134	23	25	36
Siccha	2009	61	31	34	37
Munz	2015	283	31	35	38

**Table 4:** ForCenS sample metadata description.

**Name**	**Type**	**Unit**	**Description**
*Sample_name*	*string*	*NA*	The name of the sample
*Sample_ID*	*string*	*NA*	A unique descriptor for the sample
*Error_flag*	*integer*	*NA*	A binary coded flag for the sample treatment (see Table 6)
*Device*	*string*	*NA*	Sampling device
*Latitude*	*double*	*decimal degrees*	Decimal latitude in the range of −90 (90° South) to +90 (90° North).
*Longitude*	*double*	*decimal degrees*	Decimal longitude in the range of −180 to (180° West) +180 (180° East).
*Water_depth*	*integer*	*meter*	Water depth at the sampling site
*Ocean_flag*	*integer*	*NA*	A binary coded flag denoting the ocean basin (see Table 7)
*Sample_depth_upper*	*double*	*meters*	Upper sediment depth boundary for the sample
*Sample_depth_lower*	*double*	*meters*	Lower sediment depth boundary for the sample
Sample_depth_average	*double*	*meters*	Average sediment depth for the sample
*Author*	*string*	*NA*	Author of the sample data (or compilation)
*Journal*	*string*	*NA*	Journal of the publication associated with the sample data
*Year*	*integer*	*date*	Year of the publication associated with the sample data
*Publication_doi*	*string*	*doi*	Digital Object Identifier of the sample data publication
*Resource_doi*	*string*	*doi*	Digital Object Identifier of the resource from where the sample data was retrieved
*Comment*	*string*	*NA*	Comment to sample and annotation of any modifications to the sample data
*Database_flag*	*integer*	*NA*	A binary coded flag denoting the source database of the sample (see Table 8)
*Type*	*integer*	*NA*	Variable denoting the original sample data type, 0 for relative abundances, 1 for raw count data
*Count_min*	*integer*	*individuals*	Minimum number of counted individuals per sample in the study
*Count*	*integer*	*individuals*	Number of counted individuals in the sample

**Table 5:** ForCenS database error flag description.

**Bit**	**Value**	**Description**
1	1	modified
2	2	outlier (not yet implemented)
3	4	dissolution affected (not yet implemented)
4	8	duplicate (see comment)
5	16	taxonomically incorrect (see comment)
6	32	too many unidentified (>5%)
7	64	sum of count data deviates from 100% by more than 5%
8	128	too few counted individuals (<150)
9	256	non-standard sampling device
10	512	no geographical coordinates

**Table 6:** ForCenS database constituent database flag description.

**Bit**	**Value**	**Database**	**Reference**
1	1	CLIMAP	26
2	2	Brown University Foraminiferal Database	27
3	4	ATL947	28
4	8	MARGO North Atlantic	29
5	16	MARGO South Atlantic	29
6	32	MARGO Indo-Pacific	31
7	64	MARGO Pacific	30
8	128	MARGO Mediterranean	32

**Table 7:** ForCenS sample metadata description WOA09 basin mask (Data Citation 18)

**Bit**	**Value**	**Area**
1	1	All oceans
2	2	Atlantic
3	4	North Atlantic
4	8	South Atlantic
5	16	Pacific
6	32	North Pacific
7	64	South Pacific
8	128	Indian Ocean
9	256	Southern Ocean
10	512	Arctic Ocean
11	1,024	Mediterranean Sea
12	2,048	Red Sea

**Table 8:** Results of the sequential processing of the constituent datasets of ForCenS.

	**cumulative**			**stepwise**				
	**total**	**included**	**excluded**	**duplicates**	**plain**	**position**	**name**	**manual**
CLIMAP	375	351	24	—	—	—	—	—
↳ and BUFD	1,640	1,568	72	37	32	2	3	—
↳ and ATL947	2,587	2,340	247	160	157	1	2	—
↳ and MARGO	6,360	3,637	2,723	2,075	1,912	42	111	10
↳ and Additions	6,984	4,205	2,779	—	—	—	—	—
Numbers denote the numbers of samples retained or excluded from the different databases, either cumulative or for the individual step of database merging.								

**Table 9:** Results of the individual processing of the constituent datasets of ForCenS.

	**total**	**included**	**excluded**	**numerical**	**taxonomic**	**other**	**duplicates**
CLIMAP	375	351	24	6	8	12	—
BUFD	1,265	1,254	11	1	10	—	—
ATL947	947	932	15	3	9	5	—
MARGO	3,773	3,170	603	70	46	3	486
Additions	624	568	56	43	13	—	—
Numbers denote the numbers of samples retained or excluded from the different databases. The number of samples excluded for various reasons do not add up to the total number of excluded samples because one individual sample might be flagged for exclusion due to more than one reason (taxonomically invalid and of insufficient numerical quality at the same time).							
